# Human Microbiome Alterations in Antarctic Isolated, Confined, and Extreme (ICE) Environments: A Systematic Review and Meta‐Regression

**DOI:** 10.1155/ijm/3405549

**Published:** 2026-05-06

**Authors:** Mateus S. Rumão, Marcelo T. Andrade, Róger J. Costa, Gabriel J. Santana, João P. C. Cerqueira, Gustavo S. O. Lima, Nathalia C. Garcia, Debora Heller, Cristian N. Espinosa, Rosa M. E. Arantes, Michele M. Moraes, Thiago T. Mendes

**Affiliations:** ^1^ Exercise Physiology and Health Laboratory (LAFES), Federal University of Bahia, Salvador, Bahia, Brazil, ufba.br; ^2^ Postgraduate Program in Health Sciences, Federal University of Maranhão, São Luís, Maranhão, Brazil, ufma.br; ^3^ Postgraduate Program in Medicine and Health, Federal University of Bahia, Salvador, Bahia, Brazil, ufba.br; ^4^ UT Health San Antonio, San Antonio, Texas, USA, uthscsa.edu; ^5^ Escuela de Medicina, Universidad de Magallanes, Punta Arenas, Chile, umag.cl; ^6^ Institute of Biological Sciences, Federal University of Minas Gerais, Belo Horizonte, Minas Gerais, Brazil, ufmg.br; ^7^ Department of Applied Physiology and Kinesiology, University of Florida, Gainesville, Florida, USA, ufl.edu

**Keywords:** environmental stressors, expeditionary health, microbial diversity, microbiome resilience, probiotic intervention

## Abstract

Antarctica is one of the most extreme and isolated environments on Earth, serving as a natural laboratory for studying human physiological and microbial adaptation under stress. This systematic review and meta‐regression evaluated how exposure to Antarctica’s isolated, confined, and extreme (ICE) environments impacts the human microbiome, considering environmental and behavioral factors that may modulate these alterations. Following PRISMA guidelines and being registered with PROSPERO (CRD42024558423), comprehensive searches were conducted in PubMed, Scopus, Embase, Cochrane, and LILACS without language or date restrictions. Seven studies conducted between 2014 and 2024, enrolling 77 healthy participants in total, investigated oral, gut, or skin microbiota changes under Antarctica’s ICE conditions at field camps, research stations, or shipboard environments. The findings suggest that ICE environments could induce measurable changes in microbial composition, relative abundance, and diversity across gut, oral, and skin sites, with age, type of accommodation, and sampling site emerging as significant predictors. Specifically, older individuals and those stationed at research bases exhibited greater odds of microbial alterations, while the gut microbiota showed greater stability compared to skin and oral sites. Although some studies explored the effects of probiotic supplementation and physical activity as modulators, evidence remains limited. The review highlights that environmental stressors such as confinement, duration of stay, extreme climate, diet, and physical exertion may collectively influence microbiome dynamics. These findings highlight the influence of age, accommodation type, and sampling site on microbial shifts in ICE environments, while underscoring the need for standardized longitudinal studies to clarify the biological and operational relevance of these changes.

## 1. Introduction

The human microbiome comprises trillions of microorganisms, including bacteria, viruses, and fungi, that colonize various body sites such as the gastrointestinal tract, oral cavity, skin, and other mucosal surfaces [1]. These microbial communities, referred to as microbiota [2], play a critical role in maintaining host homeostasis by regulating metabolic processes, modulating immune responses, and providing protection against pathogenic organisms [3–5]. Disruption of this symbiotic relationship, known as dysbiosis, has been linked to inflammatory diseases, infections, and metabolic disorders [6].

The gut, oral, and skin microbiomes are particularly significant due to their diverse roles in human health [1]. The gut microbiota harbors the largest microbial population and contributes to nutrient fermentation, immune modulation, and the gut–brain axis [3, 7]. Additionally, the gut microbiota has been implicated in the gut–musculoskeletal axis, influencing muscle metabolism, inflammation, and physical performance [8]. The oral microbiota, which contains the second‐largest microbial load, has been associated with inflammatory processes and is increasingly recognized for its influence on both oral and systemic health [9–12]. The cutaneous microbiota, although less abundant and less extensively studied than the gut and oral microbiota, constitutes a complex ecosystem with distinct region‐specific microbial communities across anatomical sites. Its composition varies according to factors such as humidity, pH, sebaceous secretion, gender, and hair distribution (hirsutism) and contributes to cutaneous homeostasis by modulating immunity, protecting the epidermal barrier, and inhibiting pathogens [13, 14].

The human microbiome is shaped by intrinsic factors such as genetics, age, and disease, as well as host‐extrinsic factors related to lifestyle and environmental changes, such as diet, physical activity, antibiotic use, circadian rhythms, and temperature [4, 11, 15]. Among the contexts that can foster extrinsic changes in the human microbiome, the Antarctic isolated, confined, and extreme (ICE) environment stands out.

The ICE environments, characterized by environmental stressors, limited social contact, and altered routines, are known to affect both physiological and psychological functions and may increase susceptibility to dysbiosis in exposed individuals [16, 17].

A recent systematic review examined the effects of ICE environments, including spaceflight, space analogs, and terrestrial habitats, on the gut microbiota and included one study conducted in Antarctica. The review found that these environments can affect microbial diversity and taxonomic abundance. However, data specific to Antarctica are still scarce, as the review included only a 30‐day ship‐based mission and one study conducted in Antarctic ICE conditions [18].

Within Antarctica, ICE conditions vary depending on the operational context, such as research stations, camps, or ships, each characterized by distinct structural, social, and environmental features [19]. Considering this variability, it is important to understand how specific ICE conditions in Antarctica affect human microbiome composition and which environmental or behavioral factors modulate these changes.

Therefore, this systematic review and meta‐regression was aimed at examining how exposure to the ICE environment of Antarctica affects the human microbiome and at assessing which variables, such as age, physical activity, season, expedition duration, and accommodation type (station, camp, or ship), are associated with the likelihood of microbiome alteration. The microbiome sampling site (gut, skin, or oral) was considered to account for differences between distinct microbial niches.

Based on prior evidence that age, diet, physical activity, and environmental stressors influence microbiota composition [7, 18, 20], we hypothesized that exposure to Antarctic ICE environments would be associated with a higher likelihood of microbiome alteration. We expected that these effects would be more pronounced during prolonged expeditions, during the winter season, and in station‐based accommodations, due to greater confinement and reduced environmental variation. In line with previous studies indicating age‐related declines in microbial diversity and the stabilizing effect of physical activity, we expected older individuals and those with lower physical activity levels to show greater susceptibility to microbiome alterations. Additionally, we hypothesized that the gut microbiota would exhibit greater stability compared to oral and skin microbiota, given its more buffered, host‐regulated environment.

## 2. Methods

This systematic review was conducted in accordance with the Preferred Reporting Items for Systematic Reviews and Meta‐Analyses (PRISMA) guidelines and was registered in the International Prospective Register of Systematic Reviews (PROSPERO) (registration number CRD42024558423) [21].

### 2.1. Eligibility Criteria

Studies were included if they met the following criteria: (1) primary research evaluating the human microbiome in Antarctic settings; (2) studies conducted in ICE environments such as research stations, field camps, or ship‐based missions; and (3) investigations that explored how ICE conditions may influence the human microbiome. Exclusion criteria included ongoing studies, duplicate reports, conference abstracts, and non–peer‐reviewed materials such as conference proceedings.

### 2.2. Information Sources and Search Strategy

The databases searched were Medline (PubMed), Scopus, Embase, Cochrane, and LILACS, without date or language restrictions, up to June 30, 2024. The search strategy combined Medical Subject Headings (MeSH/DeCS) and free‐text terms using Boolean operators (“AND” and “OR”). The following search string was applied: (antarctic OR antarctica) AND (microbiota OR microbiome). In addition, artificial intelligence–assisted snowballing was conducted using the ResearchRabbit platform (https://researchrabbitapp.com/) to identify additional studies through reference tracking.

The search was updated on March 2, 2025, based on the eligibility criteria established in the protocol. This update led to the identification of one additional study that met all inclusion criteria. The study underwent full screening, risk of bias assessment, and data extraction and was incorporated into the final synthesis.

### 2.3. Study Selection and Data Extraction

One reviewer (M.S.R.) conducted the initial database search and uploaded all records to Rayyan (https://new.rayyan.ai/). Two independent reviewers (M.S.R. and G.J.S.) screened titles and abstracts to identify potentially eligible studies. Full‐text screening was also conducted independently, and disagreements were resolved through discussion. When no consensus was reached, a third reviewer (J.P.C.C.) was consulted.

Data were extracted using a standardized spreadsheet in Microsoft Excel (Microsoft 365). Extracted variables included participant characteristics (biological sex, age, and nationality); study features (title, objectives, ICE environment type, and microbiome sampling site); design details (time point of sample collection and sequencing methods); and key outcomes.

### 2.4. Risk of Bias Assessment

The methodological quality and risk of bias of included studies were assessed using Cochrane tools: the Risk of Bias 2.0 (RoB 2.0) tool for randomized trials and the Risk Of Bias In Non‐randomized Studies of Exposures (ROBINS‐E) tool for observational studies. Assessments were performed independently by two reviewers (M.S.R. and R.J.C.), with disagreements resolved by a third reviewer (T.T.M.).

For randomized trials, the RoB 2.0 tool evaluated five domains: (1) bias arising from the randomization process, (2) bias due to deviations from intended interventions, (3) missing outcome data, (4) bias in measurement of outcomes, and (5) bias in selection of reported results.

For observational studies, the ROBINS‐E tool assessed seven domains: (1) bias due to confounding, (2) bias in exposure measurement, (3) bias in participant selection, (4) bias due to postexposure interventions, (5) missing data, (6) bias in outcome measurement, and (7) bias in reporting results.

All tools were applied following Cochrane guidelines. Risk of bias visualizations were generated using the RobVis web application (https://robvis.shinyapps.io/robvis/).

### 2.5. Data Synthesis and Meta‐Regression

A logistic meta‐regression was conducted to assess the association between demographic, environmental, and behavioral variables and the likelihood of microbiome alteration (binary outcome: yes = 1 and no = 0). Initial independent variables included age, physical activity (yes/no), duration of the expedition (in days), season (summer or winter), accommodation type (station, camp, or ship), and microbiome sampling site (gut, skin, or oral).

The dataset comprises 94 records from 77 unique participants across seven studies. All participant‐level data extracted and analyzed for this systematic review and meta‐regression are publicly available (DOI:https://doi.org/10.6084/m9.figshare.30068548). The dataset has been curated to ensure transparency and reproducibility and is intended to facilitate secondary analyses and methodological standardization in Antarctic microbiome research.

Model selection was guided by both statistical fit and biological plausibility. Specifically, we used the Akaike Information Criterion (AIC) to compare nested models, applying a threshold of *Δ*AIC≥2 to justify model preference. In cases where *Δ*AIC<2, variables were retained or excluded based on theoretical relevance and effect stability. Variables such as physical activity, season, expedition duration, and ship‐based accommodation were excluded from the final model based on lack of statistical significance, poor model fit (*Δ*AIC<2), or high multicollinearity in multivariable models.

We evaluated the potential nonindependence of repeated measures using generalized linear mixed‐effects models (GLMMs) with a random intercept for participant ID. As random effect variance was negligible (*σ* ^2^ < 0.6) and AIC fit did not improve substantially (67.77 vs. 69.67), fixed‐effects logistic regression was deemed the most parsimonious and robust approach for final inference (see the Supporting Information section).

All statistical analyses were conducted in R (Version 4.5.0; R Core Team, 2025) within RStudio (Version 2025.05.0+496; Posit Software, PBC). The pacman package was used for package management, readxl for data import, and dplyr and rstatix for data wrangling and descriptive summaries. The car package supported multicollinearity diagnostics, while broom and broom.mixed were used to extract model estimates and confidence intervals (CIs). GLMMs were fitted using the lme4 package. The detectseparation package was applied to identify potential separation issues, but manual inspection of diagnostics guided final decisions.

## 3. Results

### 3.1. Study Selection

**Figure 1:**
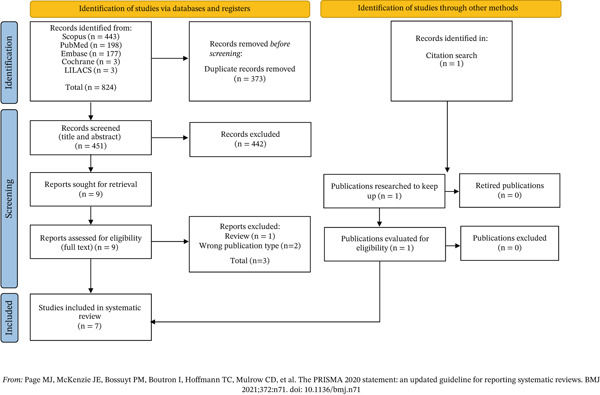
PRISMA 2020 flow diagram of the study selection process. The diagram illustrates the identification, screening, eligibility assessment, and final inclusion of studies in the systematic review based on PRISMA 2020 guidelines.

A total of 824 records were identified through database searches (PubMed = 198; Scopus = 443; Embase = 177; Cochrane = 3; LILACS = 3). After removing duplicates and screening titles and abstracts, nine full‐text articles were assessed for eligibility. Of these, two were excluded for being conference abstracts or ongoing studies without published results, and one was a prior systematic review. As part of a targeted follow‐up search based on references from eligible studies, one additional article meeting all inclusion criteria was identified. This study underwent full screening, risk of bias assessment, and data extraction and was incorporated into the final synthesis. This systematic review ultimately included seven primary studies. It is important to note that the large reduction in records after title and abstract screening was primarily due to the exclusion of studies that were not conducted on human participants (Figure 1).

### 3.2. Study Characteristics

A summary of the included studies is presented in Table 1, detailing participant demographics, type of microbiome sample collected (gut, skin, or oral), ICE environment (station, shipboard, or camp), study design and timeline, sequencing methods, and main outcomes.

**Table 1:** Summary of studies evaluating changes in the human microbiome in Antarctic isolated, confined, and extreme (ICE) environments.

Authors	Sample characteristics	Microbiota site	ICE setting	Study design	Location	Field season	Analysis method	Main findings
**Wang et al. [** **22** **]**	12 men; mean age: 42.9; nationality: Chinese	Gut and oral microbiota	Research station (367 days)	Sampling: T1 (2 months after arrival in Antarctica); T2 (2 months after T1); T3 (4 months after T1); T4 (6 months after T1); T5 (7 months postreturn)	Great Wall Station, King George Island (62°12 ^′^59 ^″^ S, 58°57 ^′^52 ^″^ W)	Summer and winter	16S rRNA sequencing	Gut microbiota underwent notable alterations during and after the expedition. Increased abundance of opportunistic pathogens, including *Citrobacter* and *Escherichia/Shigella*, persisted up to 7 months after return.
**Cameron et al. [** **23** **]**	5 men; mean age: 37; nationality: British	Oral and gut microbiota	Field (243 days)	Sampling: baseline and monthly collections throughout the 8‐month expedition	Antarctic interior, Trans‐Antarctic Winter Traverse (route)	Winter	16S rRNA sequencing	Salivary microbiota showed increases in bacterial load and diversity. No significant changes were observed in gut microbiota composition.
**Moraes et al. [** **24** **]**	5 men, 2 women; mean age: 32.3; nationality: Brazilian	Oral microbiota	Field (49 days)	Sampling: preexpedition, early‐exposure, and postexpedition collections over 46 days	Nelson Island, South Shetland (53°10 ^′^43 ^″^ S, 70°53 ^′^59 ^″^ W)	Summer	16S rRNA sequencing	Significant reduction in *Pseudomonadota* abundance (*F* = 4.280, *p* = 0.042; ES = 1.3). No significant changes were observed in other phyla.
**Srivastava et al. [** **25** **]**	19 men (placebo: 9, probiotic: 10); mean age: 29.7 and 33.4; nationality: Indian	Gut microbiota	Ship (24 days)	Sampling: baseline (predeparture) and postexpedition; randomized controlled trial with probiotic vs. placebo	Bharati Station (69°24 ^′^24 ^″^ S, 76°11 ^′^36 ^″^ E)	Summer	WGS	The probiotic group maintained gut microbiota homeostasis; the placebo group exhibited reduced diversity. Intervention: WONDERPRO probiotic supplement.
**Bhushan et al. [** **26** **]**	12 men; mean age: 32.5; nationality: Indian	Oral microbiota	Ship and station (55 days)	Sampling: baseline, midpoint, and postexpedition during Antarctic deployment	Maitri Station, Central Antarctica (70°45 ^′^52 ^″^ S, 11°44 ^′^03 ^″^ E)	Summer	16S rRNA and WGS	Oral microbiota composition changed over time. Shifts observed in *Firmicutes*, *Pseudomonadota,* and *Bacteroidetes* during the expedition.
**Sugita et al. [** **27** **]**	16 men; mean age: 43.3; nationality: Japanese	Skin microbiota	Research station (121 days)	Sampling: preexpedition (2 weeks before deployment); 3 monthly samples during stay; postexpedition (2 weeks after return)	Sør Rondane Mountains, Eastern Antarctica (~72°00 ^′^ S, 25°00 ^′^ E approx.)	Summer	qPCR	Increased colonization by *Malassezia* species was observed. Notable shifts in *M. globosa* and *M. restricta* occurred with limited access to hygiene.
**Jin et al. [** **28** **]**	6 men; mean age: 43; nationality: Japanese	Gut microbiota	Field (91 days)	Sampling: baseline in Japan; midpoint collections (monthly during 2nd, 3rd, and 4th months); postexpedition in Japan	Sør Rondane Mountains, Eastern Antarctica (~72°00 ^′^ S, 25°00 ^′^ E approx.)	Summer	T‐RFLP and qPCR	Gut microbiota changed in all participants with interindividual variability. *Bifidobacteria* levels tended to decrease during the expedition and partially recover postreturn. Three participants returned to near‐baseline profiles.

*Note:* The probiotic intervention in Srivastava et al. [25] involved WONDERPRO (LifeZen): a sachet containing 1 g of excipients (sorbitol, xylitol, maltodextrin, stabilizers, anticaking agents) and 5 × 10^9^ *C* *F* *U*/*g* of probiotic organisms, including *Lactobacillus acidophilus* (1.6 × 10^9^ *C* *F* *U*/*g*), *Lactobacillus rhamnosus* (0.8 × 10^9^ *C* *F* *U*/*g*), *Bifidobacterium longum* (0.8 × 10^9^ *C* *F* *U*/*g*), *Saccharomyces boulardii* (0.2 × 10^9^ *C* *F* *U*/*g*), and *Bacillus coagulans* (1.6 × 10^9^ *C* *F* *U*/*g*). Coordinates: S = South; E = East; W = West.

Abbreviations: 16S rRNA, 16S ribosomal RNA sequencing; qPCR, quantitative polymerase chain reaction; T‐RFLP, terminal restriction fragment length polymorphism; WGS, whole genome shotgun sequencing.

### 3.3. Risk of Bias Assessment

**Figure 2:**
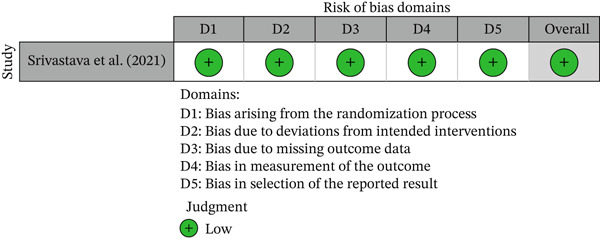
Traffic light plot summarizing risk of bias for randomized controlled trials assessed using the revised Cochrane tool (RoB 2.0). The figure illustrates domain‐level and overall risk of bias judgments for each included randomized trial. Risk levels are color‐coded as follows: green = low risk.

**Figure 3:**
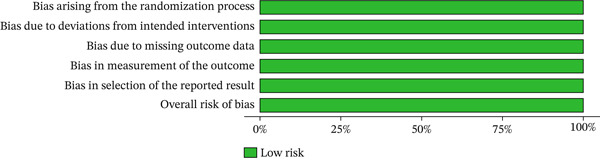
Summary plot of risk of bias assessments for randomized controlled trials using the revised Cochrane tool (RoB 2.0). The plot displays the proportion of judgments across five bias domains for each included randomized trial. Color coding represents risk levels: green = low risk; yellow = some concerns.

**Figure 4:**
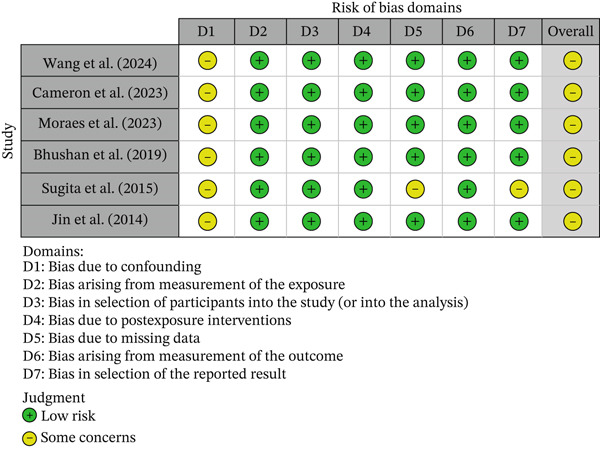
Traffic light plot summarizing risk of bias for observational studies assessed using the Risk Of Bias In Non‐randomized Studies of Exposures (ROBINS‐E) tool. The plot shows domain‐level and overall risk of bias judgments for each included nonrandomized study. Risk levels are color‐coded as follows: green = low risk; yellow = some concerns.

**Figure 5:**
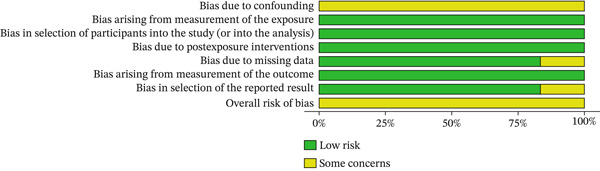
Summary plot of risk of bias assessments for observational studies using the ROBINS‐E tool. The figure shows the proportion of studies rated for each of the seven ROBINS‐E domains, as well as the overall risk of bias. Green bars indicate low risk; yellow bars indicate some concerns.

One study was a randomized trials and was assessed using the RoB 2.0 tool, while the remaining six observational studies were assessed using the ROBINS‐E tool. The methodological quality of the included studies, based on the risk of bias assessment, is presented through a traffic light plot and a summary plot for the randomized trials (Figures 2 and 3) and for the observational studies (Figures 4 and 5). Most studies were rated as having “some concerns” or “low risk of bias,” though two observational studies showed “serious” concerns in the domain of confounding.

### 3.4. Meta‐Regression

The final logistic meta‐regression model identified age and accommodation type as significant predictors of microbiome alteration during Antarctic expeditions. In addition, the microbiome sampling site was associated with differences in the likelihood of alteration, reflecting distinct microbial niches (gut, skin, and oral). Age was positively associated with the odds of microbiome alteration: for each additional year, the likelihood increased by 49% (OR = 1.49; 95% CI: 1.23–1.89; *p* < 0.001), suggesting decreased microbiome resilience with aging under ICE conditions.

Accommodation type was also a strong predictor. Participants based in field camps had significantly lower odds of microbiome alteration compared to those at research stations (OR = 0.021; 95% CI: 0.002–0.118; *p* < 0.001), potentially reflecting differences in exposure duration, activity levels, or environmental stress. The microbiome sampling site was associated with differences in the likelihood of microbiome alteration. Compared to skin samples, gut microbiota showed greater stability, with lower odds of alteration (OR = 0.068; 95% CI: 0.010–0.294; *p* = 0.001), reflecting distinct microbial niches and consistent with prior evidence of intestinal microbiome resilience.

As a sensitivity analysis, an alternative model replacing gut with oral microbiota sampling was also fitted. This model yielded similar results, identifying a strong positive association between oral sampling and microbiome alteration (OR = 15.2; 95% CI: 3.43–104.0; *p* = 0.001), with comparable model fit (AIC = 67.99 vs. 67.77). Nonetheless, the gut‐based model was retained due to its slightly superior fit and biological plausibility.

Other tested predictors were excluded from the final model due to lack of statistical significance or poorer model fit. These included season (winter vs. summer) (OR = 1.56; 95% CI: 0.25–10.7; *p* = 0.639), duration of expedition (OR = 1.00; 95% CI: 0.98–1.03; *p* = 0.812), and ship‐based accommodation (OR = 1.39; 95% CI: 0.42–4.63; *p* = 0.589). Although physical activity was strongly associated with reduced odds of microbiome alteration in simplified models (OR = 0.056; 95% CI: 0.013–0.199; *p* < 0.001), its inclusion introduced multicollinearity and model instability. It was therefore excluded from the final model for reasons of parsimony and interpretability.

## 4. Discussion

Drawing on 77 participant records from seven primary studies (2014–2024), we examined microbial composition in the gut, oral, and skin microbiota of healthy volunteers—predominantly researchers and military personnel. Expedition lengths ranged from 24 to 367 days and were carried out across three ICE contexts: field camps, research stations, and ship‐based missions.

Environmental and behavioral factors inherent to ICE conditions were associated with microbial dynamics. Our meta‐regression identified age and accommodation type as significant predictors of microbiome alteration. In addition, the microbiome sampling site was associated with differences in the likelihood of alteration, reflecting distinct microbial niches. Older individuals were substantially more likely to experience microbiome changes, with each additional year of age increasing the odds of alteration by 49% (OR = 1.49; 95% CI: 1.23–1.89; p <0.001). This finding aligns with previous literature reinforcing that age‐related reductions in microbiome resilience may be driven by factors such as immunosenescence and chronic low‐grade inflammation [20].

Moreover, participants in field camps were markedly less likely to exhibit microbiome alterations compared to those at research stations (OR = 0.021; 95% CI: 0.002–0.118; *p* < 0.001). This result was unexpected, since ICE conditions in field camps are considered among the most extreme—characterized by severe cold, high light exposure, and limited infrastructure for basic human needs [19]. One possible explanation is that field‐based missions generally involve shorter exposure durations than station‐based missions, although this may vary across studies, along with the elevated physical demands of field activities. These findings highlight the need for further investigation into the environmental and operational characteristics of field and station settings that may differentially influence microbiome dynamics. Both factors are known to influence microbiome diversity and metabolic regulation in other populations [29, 30]. In our analysis, physical activity was strongly associated with reduced odds of microbiome alteration in simplified models (OR = 0.056; 95% CI: 0.013–0.199; *p* < 0.001), supporting its potential protective role. However, due to its high collinearity with accommodation type and microbiota site, its inclusion compromised model stability, and it was excluded from the final model. Still, its significance in preliminary models is consistent with Cameron et al. [23] and Moraes et al. [24] and warrants targeted investigation in future studies designed to isolate behavioral effects.

Differences in microbiome stability were observed across sampling sites, reflecting distinct microbial ecosystems. Gut microbiota appeared more stable, whereas skin microbiota showed greater variability under ICE camping conditions, which may be associated with environmental and hygiene‐related factors [27]. Oral microbiota, by contrast, showed high diversity and marked sensitivity to environmental factors [31]. These findings align with evidence from other confined environments, including the MARS500 mission [32]. This robustness may relate to the gut’s buffered and host‐regulated ecosystem. In contrast, the oral microbiota appeared particularly sensitive to ICE‐related stress. The study by Bhushan et al. [26] reported pronounced phylum‐level shifts, including increased *Pseudomonas* and *Lactobacillus* abundance, possibly reflecting mucosal inflammation or hygiene‐related changes. An alternative version of our final model, replacing intestinal with oral microbiota, confirmed this sensitivity: oral microbiota was associated with a 15‐fold increase in the odds of alteration (OR = 15.2; 95% CI: 3.43–104.0; *p* = 0.001), with comparable model fit (AIC = 67.99 vs. 67.77). However, the intestinal microbiota model was retained as the primary model due to its slightly better fit and theoretical relevance.

Ship‐based expeditions, characterized by environmental and physiological stressors such as confinement, circadian disruption, and motion sickness, were evaluated in our synthesis but not retained in the final model due to statistical nonsignificance (OR = 1.39; 95% CI: 0.42–4.63; *p* = 0.589). Nonetheless, their biological relevance is supported by studies such as Srivastava et al. [25], who found that gut microbial diversity declined during sea transit but was preserved in participants receiving a probiotic supplement WONDERPRO (LifeZen). Although not predictive in our meta‐regression, our data suggest that microbiome‐targeted countermeasures may be valuable for future maritime expeditions, which involve extreme confinement and strenuous working conditions, especially for individuals responsible for vessel maintenance or in other confined locations, and warrant specific investigation.

The potential impact of extended ICE exposure on the human microbiome was assessed in our models, but expedition duration did not emerge as an independent predictor (OR = 1.00; 95% CI: 0.98–1.03; *p* = 0.812). This apparent null finding contrasts with evidence from Wang et al. [22], who documented persistent intestinal colonization by opportunistic pathogens such as *Citrobacter* and *Escherichia/Shigella* up to 7 months after completion of a 12‐month station‐based expedition. Conversely, Jin et al. [28] observed reversion of gut microbiota composition toward baseline after a 3‐month field camp, suggesting that recovery trajectories may vary with exposure length and context. The lack of association in our analysis likely reflects the heterogeneity of reporting across studies and the limited granularity of duration data, rather than the absence of a biological effect. Thus, while duration could not be confirmed as a predictor in this synthesis, cumulative evidence underscores the plausibility of long‐term microbiome disruption in missions exceeding several months—an issue warranting systematic, prospective evaluation.

Despite these insights, the evidence base remains constrained. Given the unique logistical and ethical challenges of conducting research in Antarctica, only seven studies met the eligibility criteria. Even within this limited corpus, key associations emerged as statistically significant and remained consistent across models. Some potentially relevant variables could not be examined due to collinearity, instability, or data imbalance. For example, supplementation status and nationality were not reliably assessed, and biological sex was markedly underrepresented, with only two female participants across all studies. This imbalance mirrors a broader trend in ICE research and underscores the urgency of greater inclusion and sex‐specific analyses [33].

Analytical considerations also temper the interpretation of our findings. Logistic meta‐regression was appropriate for exploring predictors across heterogeneous studies, yet the small number of primary studies increases the risk of overfitting and unstable estimates, even when restricted to biologically plausible variables. Substantial methodological heterogeneity further limits comparability, including differences in sequencing platforms, operational taxonomic unit (OTU) thresholds, and definitions of microbiome “alteration.”

In summary, this study has limitations that should be considered. The small number of included studies limits statistical power and the generalizability of the findings. In addition, methodological heterogeneity, data imbalance, collinearity among variables, and variation in exposure‐related factors across studies limit comparability and causal interpretation of the results. These issues largely reflect structural constraints in Antarctic microbiome research rather than just weaknesses of the present synthesis. Coordinated international efforts, ideally through a dedicated microbiome consortium, could harmonize data, enable individual participant data (IPD) meta‐analyses, and support multicenter longitudinal studies to map microbiome adaptation in ICE environments with greater precision.

The Antarctic ICE environment constitutes a natural laboratory for studying host–microbe interactions under extreme and confined conditions. Our findings may support the inclusion of microbiome monitoring in health surveillance protocols in extreme environments. For clinicians and expeditionary medical teams, this information may contribute to the development of preventive strategies, such as nutritional management and hygiene practices, aimed at maintaining microbial stability during prolonged missions. Randomized trials are needed to systematically test interventions involving structured exercise, dietary modulation, and probiotic use.

## 5. Conclusion

Exposure to the ICE conditions in Antarctica is associated with measurable alterations in the human microbiome, particularly among older individuals and depending on accommodation type, while differences across sampling sites reflect distinct microbial niches. The gut microbiota appears more resilient than skin and oral niches, and field camp environments may confer partial protection. Beyond individual findings, this systematic review and meta‐regression provided the first integrated evidence base on microbial vulnerability and adaptation in Antarctica, offering a framework to guide future experimental studies and microbiome‐targeted interventions in extreme environments.

## Funding

Funding was provided by the Conselho Nacional de Desenvolvimento Científico e Tecnológico (CNPq), the Ministério da Ciência, Tecnologia e Inovação (MCTI), the Fundo Nacional de Desenvolvimento Científico e Tecnológico (FNDCT), and the Programa Antártico Brasileiro (PROANTAR) under grants CNPq/MCTI/FNDCT 440932/2023‐8, CNPq/MCTI 408740/2023‐0, and MCTI/CNPq 404878/2024‐5, as well as by the Fundação de Amparo à Pesquisa do Estado da Bahia (FAPESB). R.M.E.A. received a research fellowship from the CNPq under grant PQ 311976/2021‐2.

## Conflicts of Interest

The authors declare no conflicts of interest.

## Supporting information


**Supporting Information** Additional supporting information can be found online in the Supporting Information section. S1: DOI.

## Data Availability

The data that support the findings of this study are openly available in Figshare at 10.6084/m9.figshare.30068548.v1.
