# Supplementary material for: Human Microbiome Alterations in Antarctic Isolated, Confined, and Extreme (ICE) Environments: A Systematic Review and Meta‐Regression
Source: Int J Microbiol. 2026 May 6;2026:3405549. doi: 10.1155/ijm/3405549 (PMC13149223; doi:10.1155/ijm/3405549)
Supplement: Supplementary file 1 — Supporting Information Additional supporting information can be found online in the Supporting Information section. S1: DOI. [file IJM-2026-3405549-s001.docx]

**Supplementary Material: Assessment of Logistic Regression Assumptions**

**Introduction**

To ensure the validity and interpretability of the logistic regression models used in this study (Models 4a and 4b), a comprehensive evaluation of the core assumptions of binary logistic regression was conducted. Model 4a included age, accommodation type (field camp vs. research station), and gut microbiota sampling; Model 4b was an alternative specification using oral microbiota sampling instead.

**1. Binary Outcome Variable**

The dependent variable, microbiome alteration, was binary (0 = no alteration, 1 = alteration) and explicitly encoded as a factor in R, with 0 as the reference category. This satisfies the fundamental requirement for binary logistic regression.

**2. Absence of Multicollinearity**

Variance Inflation Factors (VIFs) were calculated for all model predictors. All values were well below the conservative threshold of 5, indicating no problematic multicollinearity. In Model 4a, VIFs were 2.04 (age), 2.37 (accommodation), and 1.53 (gut microbiota); in Model 4b, corresponding values were 1.92, 2.25, and 1.54 (oral microbiota).

**3. Linearity of the Logit for Continuous Predictors**

The relationship between the continuous predictor (age) and the logit of the outcome was assessed using component-plus-residual plots (crPlots, car package in R). The smoothed residual lines closely followed the expected linear reference lines, indicating that the assumption of linearity was reasonably satisfied in both models.


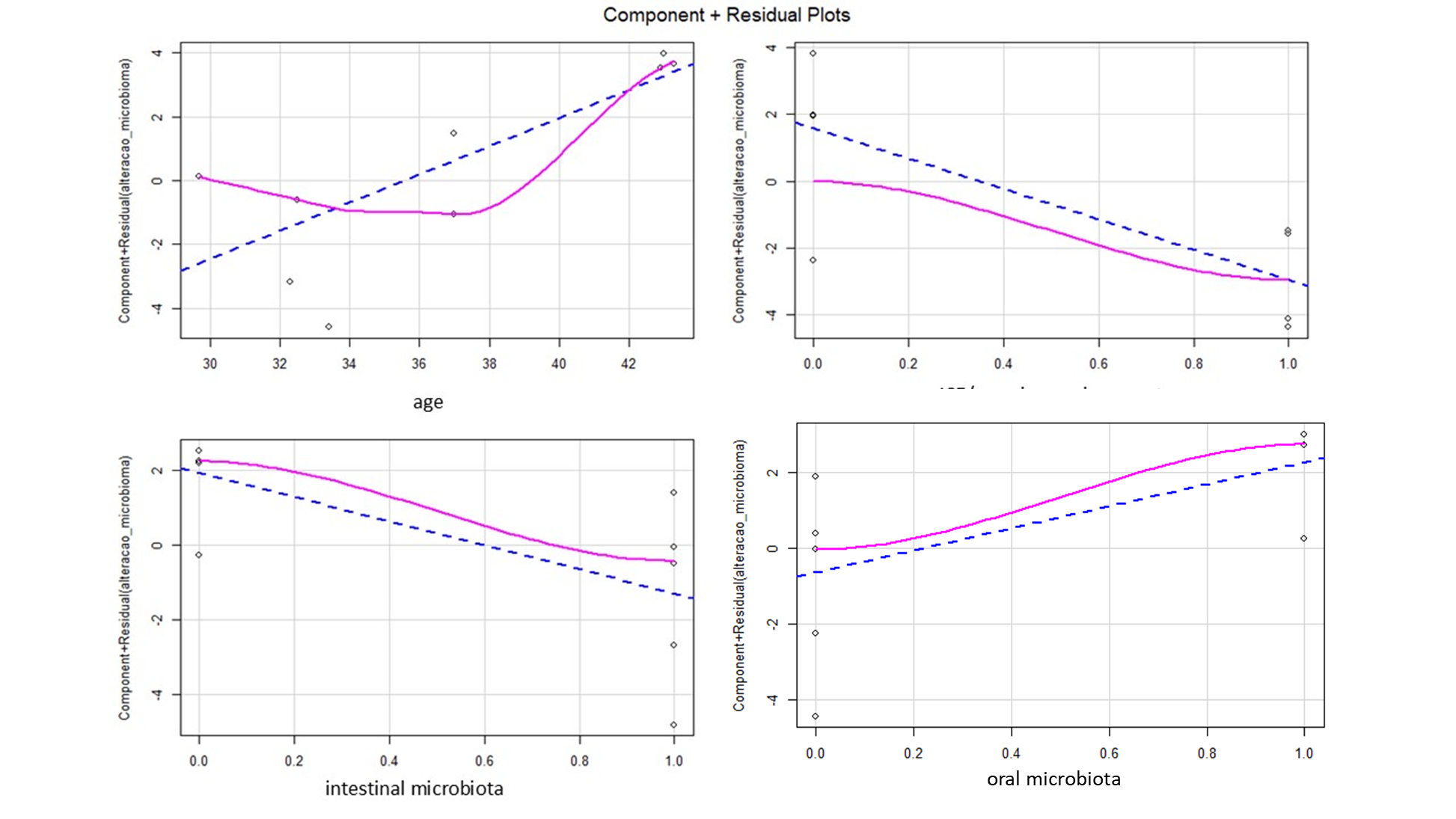


Supplementary Figure 1. Component-plus-residual plots for covariates included in the final logistic models. Top row: age (left) and accommodation type (right), common to both Model 4a (with intestinal microbiota) and Model 4b (with oral microbiota). Bottom row: intestinal microbiota (left, Model 4a) and oral microbiota (right, Model 4b). The magenta lines represent smoothed residuals; blue dashed lines indicate the expected linear relationship with the logit. No substantial deviations from linearity were observed for any variable.

**4. Absence of Complete or Quasi-Complete Separation**

No evidence of separation was observed. All coefficients were finite and interpretable, standard errors were low (all < 1.0), and no convergence warnings were issued. These diagnostics confirm the absence of complete or quasi-complete separation in the data.

**5. Model Convergence and Estimation Stability**

Both models converged successfully within six Fisher scoring iterations. Model fit was strong, with residual deviance reduced from 102.29 (null) to 59.77 (Model 4a) and 59.99 (Model 4b). AIC values were within acceptable range (Model 4a: 67.77; Model 4b: 67.99), confirming stability and reliability of estimation.

**Conclusion**

All core assumptions of binary logistic regression were adequately met for the final models. Diagnostic checks confirmed the robustness, interpretability, and statistical validity of the primary model (Model 4a) and its alternative specification (Model 4b).
